# Molecular evolution of dengue virus types 1 and 4 in Korean travelers

**DOI:** 10.1007/s00705-021-04973-8

**Published:** 2021-02-11

**Authors:** Eun-Ha Hwang, Green Kim, Hoyin Chung, Hanseul Oh, Jong-Hwan Park, Gyeung Haeng Hur, JungJoo Hong, Bon-Sang Koo

**Affiliations:** 1 National Primate Research Center, Korea Research Institute of Bioscience and Biotechnology, Cheongju, Republic of Korea; 2 Laboratory of Animal Medicine, College of Veterinary Medicine, Chonnam National University, Gwangju, Republic of Korea; 3 Department of Microbiology, College of Natural Sciences, Chungbuk National University, Cheongju, Republic of Korea; 4 Agency for Defense Development, Daejeon, Republic of Korea

## Abstract

Dengue virus (DV) is a mosquito-borne virus that is endemic to many tropical and subtropical areas. Recently, the annual incidence of DV infection has increased worldwide, including in Korea, due to global warming and increased global travel. We therefore sought to characterize the molecular and evolutionary features of DV-1 and DV-4 isolated from Korean overseas travelers. We used phylogenetic analysis based on the full coding region to classify isolates of DV-1 in Korea into genotype I (43251, KP406802), genotype IV (KP406803), and genotype V (KP406801). In addition, we found that strains of DV-4 belonged to genotype I (KP406806) and genotype II (43257). Evidence of positive selection in DV-1 strains was identified in the C, prM, NS2A, and NS5 proteins, whereas DV-4 showed positive selection only in the non-structural proteins NS2A, NS3, and NS5. The substitution rates per site per year were 5.58 × 10^-4^ and 6.72 × 10^-4^ for DV-1 and DV-4, respectively, and the time of the most recent common ancestor was determined using the Bayesian skyline coalescent method. In this study, the molecular, phylogenetic, and evolutionary characteristics of Korean DV-1 and DV-4 isolates were evaluated for the first time.

**Supplementary Information:**

The online version contains supplementary material available at 10.1007/s00705-021-04973-8.

## Introduction

Dengue fever is a major mosquito-borne viral illness that causes significant public health problems in subtropical and tropical regions of the world [1]. Dengue virus (DV) is commonly transmitted among humans by two mosquito vectors, *Aedes aegypti* and *Aedes albopictus* [2]. Global warming and urbanization have fueled the population growth of these vectors and broadened their habitat. During the past three decades, the incidence of dengue fever has increased by more than 300% in more than 100 countries [3–5]. There are 2.9 million dengue infections and 5,906 deaths reported in Southeast Asia each year [6]. For this reason, the World Health Organization has designated dengue infection as one of the top 17 tropical diseases [7].

DV, a member of the family *Flaviviridae*, has a single-stranded positive-sense RNA genome that encodes three structural proteins (capsid [C], pre-membrane [prM], envelope [E]) and seven non-structural proteins (non-structural [NS] 1, NS2A, NS2B, NS3, NS4A, NS4B, NS5) [1]. DVs are classified into four distinct serotypes (DV-1, DV-2, DV-3, and DV-4) and subdivided into several genotypes within each serotype. Significantly high genetic variation is commonly observed in the DV genome, with 60–70% amino acid sequence identity shared between serotypes [2]. This high genetic variability is closely related to the highly variable pathogenicity and transmissibility of DV [8].

Dengue has a wide spectrum of clinical severity, from asymptomatic through a self-limiting febrile illness (dengue fever) to life-threatening severe infections such as dengue hemorrhagic fever (DHF) and dengue shock syndrome (DSS). The mechanisms underlying severe dengue infections are not completely understood, but an antibody-dependent enhancement phenomenon and infection with particularly virulent virus strains are known to correlate with severe dengue infection [9–11]. Specific amino acid changes in DV proteins are closely associated with altered virulence. For instance, mutation of amino acids 124 and 128 of the E protein, amino acid 64 of NS2A, or amino acid 55 of NS2B can increases DV virulence [11, 12].

Since the first case of dengue fever was reported in South Korea in 1995, DV infections have been increasingly reported by travelers returning from dengue-endemic countries [13]. *A. albopictus* was recently identified as a mosquito vector for dengue infection in Korea. The expanding distribution of this mosquito in urban areas increases the likelihood of outbreaks in Korea [14–16]. Indeed, autochthonous dengue infections have been reported in Japan and some European countries at approximately the same latitudes as in Korea. DV-1 and DV-4 have been reported to cause severe dengue fever at a high rate of incidence in Asian countries [17, 18]. Therefore, surveillance of these serotypes is necessary, but information on Korean DVs is very limited. For this report, we performed molecular and evolutionary analysis of DV-1 and DV-4.

## Materials and methods

### Viruses and sequencing

Isolates of DV-1 and DV-4 (NCCP43251, NCCP43257) were obtained from the National Culture Collection for Pathogens (Cheongju, Korea). These viruses were isolated from serum samples from Korean overseas travelers (Table 1). The virus was cultivated on a monolayer of Vero E6 cells (ATCC CRL-1586) for propagation in minimal essential medium (Gibco, Life Technologies, NY, USA) containing 2% fetal bovine serum and 1% penicillin (100 IU/ml)/streptomycin (100 μg/ml) (Gibco) at 37 ℃ and 5% CO_2_. Viral RNA was extracted from the supernatant of the DV-infected Vero E6 cells using a QIAamp Viral RNA Mini Kit (QIAGEN, CA, USA) according to the manufacturer's instructions. Reverse transcription (RT)-PCR was performed using a QIAGEN OneStep RT-PCR Kit with modified PCR primer sets targeting the full genome sequences of DV-1 and DV-4 (Supplementary Table S1) [19]. The PCR conditions as follows: reverse transcription at 45 ℃ for 30 minutes and denaturation at 95 ℃ for 15 minutes, followed by 40 cycles of amplification (94 ℃ for 10 seconds, 46 ℃ for 30 seconds, and 68 ℃ for 3 minutes), and then a final elongation step at 68 ℃ for 10 minutes. The final amplicons, stained with SYBR^®^ Safe DNA Gel Stain (Invitrogen, USA), were visualized on 1.6% agarose gels with UV transillumination and purified using an Expin Gel SV kit (GeneAll, Seoul, Korea). The purified products were then sequenced by the Sanger method using an ABI 3730XL System (Macrogen, Seoul, Korea). All procedures were conducted in a Biosafety Level 2 laboratory.

**Table 1:** Information about the DV-1 and DV-4 strains analyzed in this study

Type	Strain	Isolation year	Travel location	Source	Country of isolation
DV-1	KP406802	2015	Indonesia	Human serum	South Korea
	43251	2011	Indonesia	Human serum	South Korea
	KP406803	2006	Philippines	Human serum	South Korea
	KP406801	2004	Singapore	Human serum	South Korea
DV-4	43257	2010	Philippines	Human serum	South Korea
	KP406806	-	-	Human serum	South Korea

### Phylogenetic and sequence analysis

The nucleotide sequences were trimmed using Bioedit software, version 7.0.5.3., and assembled using CLC Genomics Workbench 12.0 (QIAGEN). Multiple alignments of the complete coding regions were performed with DV reference genes (97 DV-1 and 57 DV-4), including previously reported Korean isolates, using the CLUSTAL W method. Maximum-likelihood phylogenetic analysis of DV-1 and DV-4 was performed using the Tamura Nei model with gamma distributed rates and 1,000 bootstrap replicates in Molecular Evolutionary Genetics Analysis (MEGA), version X [20]. The percentages of nucleotide and amino acid sequence identity among Korean isolates were calculated as pairwise *p*-distances.

### Bayesian evolutionary analysis

The rate of nucleotide substitutions per site per year and the time to the most recent common ancestor (TMRCA) of 70 DV-1 and 54 DV-4 strains (differentiated based on their E protein sequences) was estimated using the Bayesian Markov chain Monte Carlo (MCMC) approach as implemented in the BEAST 2 package. Sequences with low quality or more than 99% identity as well as recombinant sequences were excluded from this analysis. The best-fit substitution model was selected using the Akaike information criterion and the Bayesian information criterion as implemented in CLC Genomics Workbench 12.0. The GTR + G + I model (general time-reversible model with gamma-distributed rates of variation among sites and a proportion of invariable sites) was found to be the best-fit model for the DV-1 and DV-4 datasets. Three independent MCMC analyses, each with 60,000,000 steps, were performed using the uncorrelated relaxed molecular clock with a Bayesian skyline coalescent prior, and they were combined with a burn-in value set to 10% generations using Log Combiner 2.5.2. The convergence of the chain was evaluated and viewed using Tracer version 1.7.1 (http://tree.bio.ed.ac.uk/software/tracer/). Effective sample size (ESS) values greater than 200 indicated a sufficient level of sampling. The combined trees were annotated using Tree Annotator v.1.8.2 and visualized in Figtree 1.4.2.

### Selection pressure analysis

Selection pressure was evaluated for 101 DV-1 and 59 DV-4 strains by different methods, using the datamonkey web server and the HyPhy package [21]. Sequences with ambiguous characteristics or high similarity (> 99% identity) were excluded from this analysis. The ratio of synonymous to non-synonymous (ω ratio) changes was calculated using the single-likelihood ancestor, fixed-effects likelihood, mixed-effects model of evolution, and fast unconstrained Bayesian approximation methods [22, 23]. Selection pressure analysis was performed based on the full coding regions of the structural (C, prM, and E) and non-structural (NS1, NS2A, NS2B, NS3, NS4A, NS4B, and NS5) genes. Low ω ratios (0.060-0.081) were considered an indication of negative selection. Positive and negative selection at each site were analyzed based on statistical significance (*p*-value < 0.1 or posterior probability < 0.9) by at least two methods.

## Results

### Sequencing and phylogenetic analysis

**Fig. 1:**
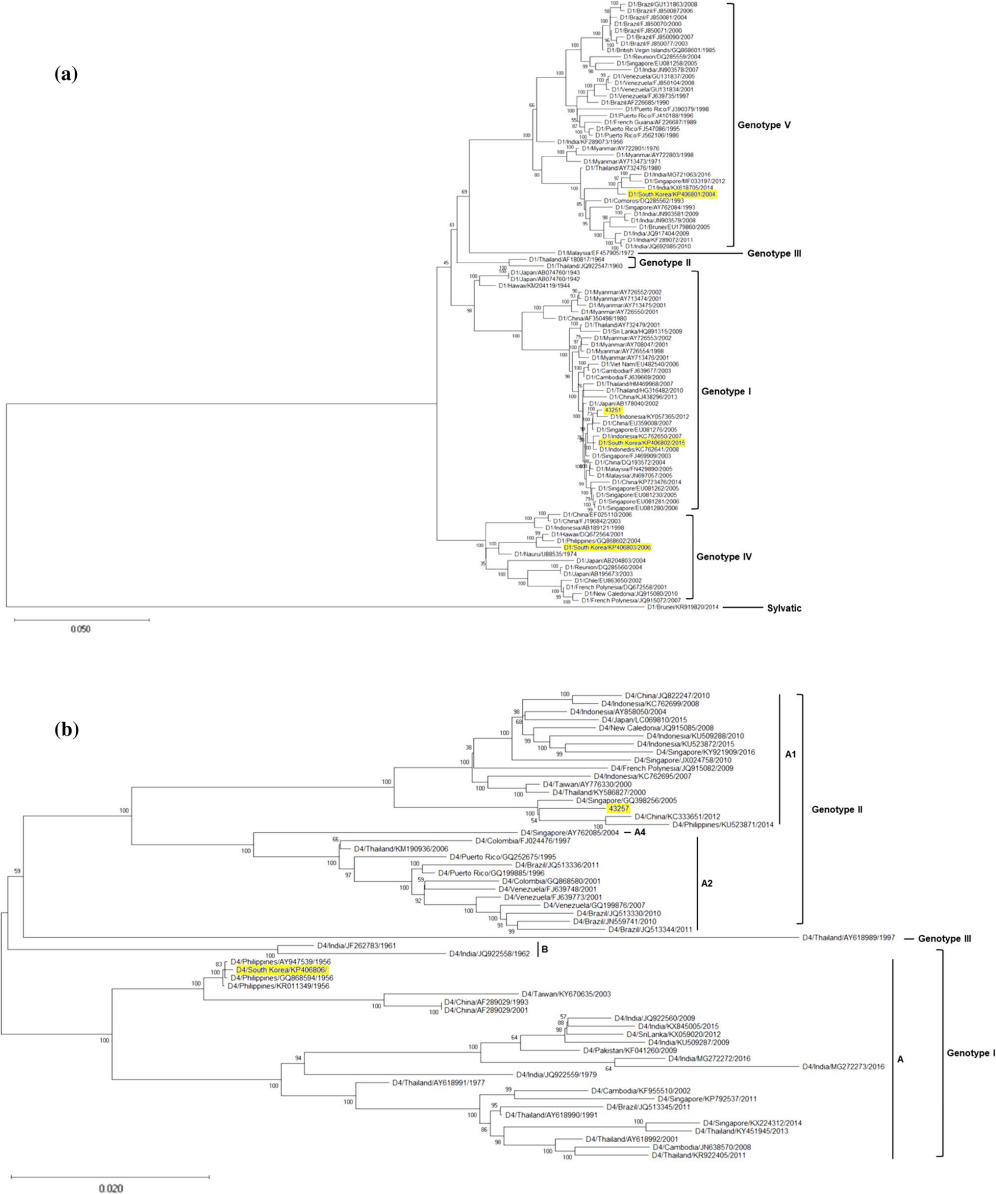
Phylogenetic analysis of DV-1 and DV-4 using the maximum-likelihood method. The trees were constructed in MEGA-X using nucleotide sequences of the complete coding region. The percentage of bootstrap support determined from 1,000 replicates is shown for key nodes. The operational taxonomic unit (OTU) label for each strain consists of four parts separated by forward slashes (‘/’): serotype/country of collection/GenBank accession number/year of collection. The scale is the number of nucleotide substitutions per site along the branch. (a) DV-1, (b) DV-4

The full coding sequences of the Korean DV-1 (43251; 10,179 nucleotides) and DV-4 (43257; 10,163 nucleotides) isolates were determined, and the serotypes of those viruses were confirmed by NCBI BLAST analysis. The phylogenetic analysis of the DV-1 and DV-4 strains was performed based on the full coding region (Fig. 1a and b). The DV-1 isolates from Korea fit into three of five previously defined genotypes: genotype I (43251, KP406802), genotype IV (KP406803), and genotype V (KP406801), based on 8.6–9.5% nucleotide sequence divergence. In addition, the DV-4 strains were classified into genotype I (KP406806) and genotype II (43257) based on 5.7% nucleotide sequence divergence. The strains most similar to our Korean isolates were DV-1 Singapore 2005 (EU081276, 99.5%) to 43251, DV-1 Indonesia 2008 (KC762641, 99.7%) to KP406802, DV-1 Singapore 2012 (MF033197, 98.7%) to KP406801, DV-1 Hawaii 2001 (DQ672564, 98.5%) to KP406803, DV-4 Singapore 2005 (GQ398256, 98.9%) to 43257, and DV-4 Philippines 1956 (AY947539, 99.9%) to KP406806. All sequences files are available from the GenBank database (accession numbers MT597439 and MT459980).

### Sequence analysis

Among the Korean DV-1 isolates, the nucleotide and amino acid sequence identity ranged from 90.5% to 98.8% and 96.4% to 99.4%, respectively. The nucleotide and amino acid sequence identity among the DV-4 isolates was 94.3% and 96.8%, respectively (Table 2). The amino acid sequence for each open reading frame of the Korean DV-1 and DV-4 isolates was compared with that of the most similar strain and the standard strain of each type (Tables 3 and 4). The region of the DV-1 isolates that was most variable, based on amino acid sequence comparisons (95.8–96.4% identity) to EU848545 (the DV-1 standard strain), was in the prM protein. Compared with the most similar strain of each DV-1 isolate, the KP406801 strain exhibited the lowest overall sequence similarity (95.4–99.2% identity). The region of the DV-4 isolates that differed the most from AF326573 (the DV-4 standard strain) was in the NS2A and NS1 proteins (95.4% and 95.5% identity, respectively). However, amino acid changes in the motifs related to virulence and viability [24, 25] were not observed in either DV-1 or DV-4 (Supplementary Tables S2 and S3).

**Table 2:** Nucleotide and amino acid sequence identity of the envelope genes and proteins of DV-1 and DV-4

	Sequence identity (%)					
Strain	KP406802	43251	KP406803	KP406801	43257	KP406806
KP406802		98.79	91.25	90.57	65.21	64.67
43251	99.39		91.45	90.51	65.08	64.41
KP406803	97.37	91.17		90.77	64.27	63.40
KP406801	96.57	96.36	97.37		64.00	63.20
43257	63.84	63.43	63.64	64.85		94.34
KP406806	62.83	62.42	62.42	63.23	96.77	

The values below the diagonal spaces indicate amino acid sequence identity (%), and the values above the diagonal spaces indicate nucleotide sequence identity (%).

**Table 3:** Amino acid substitutions in DV-1 compared to the most similar strains and standard virus

Strain	Reference strain		Structural proteins				Non-structural proteins					
		No. of substitutions (% identity)	C (114)	prM (166)	E (495)	NS1 (352)	NS2A (218)	NS2B (130)	NS3 (619)	NS4A (127)	NS4B (249)	NS5 (899)
43251	EU848545^a^		4	7	10	10	3	2	10	2	4	12
			(96.49)	(95.78)	(97.98)	(97.16)	(98.62)	(98.46)	(98.38)	(98.43)	(98.39)	(98.67)
	EU081276^b^		0	0	2	0	1	0	0	1	1	6
			(100)	(100)	(99.60)	(100)	(99.54)	(100)	(100)	(99.21)	(99.60)	(99.33)
KP406801	EU848545		4	6	10	10	4	2	10	2	4	14
			(96.49)	(96.39)	(97.98)	(97.16)	(98.17)	(98.46)	(98.38)	(98.43)	(98.39)	(98.44)
	MF033197		5	4	13	9	10	1	11	4	5	24
			(95.61)	(97.59)	(97.37)	(97.44)	(95.41)	(99.23)	(98.22)	(96.85)	(97.99)	(97.33)
KP406802	EU848545		4	7	10	10	3	2	11	2	3	11
			(96.49)	(95.78)	(97.98)	(97.16)	(98.62)	(98.46)	(98.22)	(98.43)	(98.80)	(98.78)
	KC762641		0	2	1	0	0	0	0	0	0	0
			(100)	(98.80)	(99.80)	(100)	(100)	(100)	(100)	(100)	(100)	(100)
KP406803	EU848545		4	7	7	11	8	3	10	2	4	10
			(96.49)	(95.78)	(98.59)	(96.88)	(96.33)	(97.69)	(98.38)	(98.43)	(98.39)	(98.89)
	DQ472564		1	1	1	0	0	0	1	0	1	1
			(99.12)	(99.40)	(99.80)	(100)	(100)	(100)	(99.84)	(100)	(99.60)	(99.89)

^a^ DV-1 standard strain

^b^ the most similar DV-1 to each Korean isolate

**Table 4:** Amino acid substitutions in DV-4 compared to the most similar strains and standard virus

Strain	Reference strain		Structural proteins				Non-structural proteins					
		No. of substitutions (% identity)	C (114)	prM (166)	E (495)	NS1 (352)	NS2A (218)	NS2B (130)	NS3 (618)	NS4A (127)	NS4B (245)	NS5 (900)
43257	AF326573^a^		4	0	5	5	10	0	6	0	5	20
			(96.49	(100)	(98.99)	(98.58)	(95.41)	(100)	(99.03)	(100)	(97.99)	(97.78)
	GQ398256^b^		1	3	3	7	3	1	2	3	0	7
			(99.12	(98.19)	(99.39)	(98.01)	(98.62)	(99.23)	(99.68)	(97.64)	(100)	(99.22)
KP406806	AF326573		3	4	14	16	7	4	8	3	3	8
			(97.37	(97.59)	(97.17)	(95.45)	(96.79)	(96.92)	(98.71)	(97.64)	(98.80)	(99.11)
	AY947539		1	4	2	1	0	1	0	1	1	0
			(99.12	(97.59)	(99.60)	(99.72)	(100)	(99.23)	(100)	(99.21)	(99.60)	(100)

^a^ DV-4 standard strain

^b^ the most similar DV-4 to each Korean isolate

C, capsid; prM, pre-membrane; E, envelope; NS, non-structural

### Bayesian evolutionary analysis

**Fig. 2:**
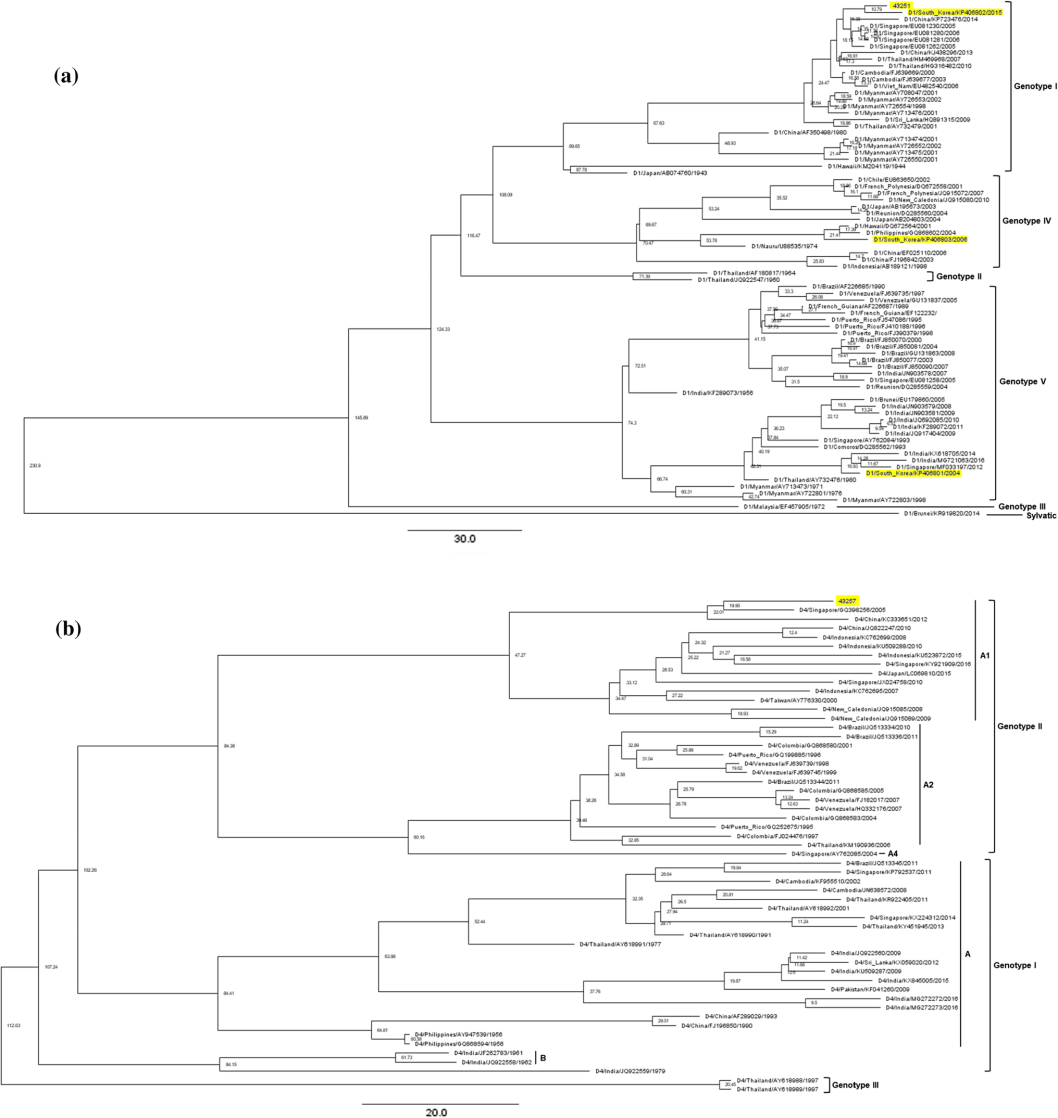
Bayesian evolutionary analysis of DV-1 and DV-4 in Korea. The relationships of DV-1 and DV-4 strains were inferred based on complete E gene nucleotide sequences using a relaxed uncorrelated lognormal molecular clock with a Bayesian skyline coalescent prior. The Korean DV sequences from the present study are indicated in yellow. The estimated 95% HDP values for the TMRCA are indicated at the node. Strains are labeled as follows: serotype/country of collection/GenBank accession number/year of collection for each strain. (a) DV-1, (b) DV-4

Rates of nucleotide substitution and TMRCA with ESS values above 200 were determined for the E genes of 70 DV-1 strains and 54 DV-4 strains (Fig. 2a and b). The number of nucleotide substitutions per site per year in the DV-1 strains was 5.58 × 10^-4^ (95% high probability density [HPD] interval: 4.00 × 10^-4^ to 7.13 × 10^-4^), and the rate in the DV-4 strains was 6.72 × 10^-4^ (95% HPD interval: 5.52 × 10^-4^ to 7.94 × 10^-4^). The TMRCA of each epidemic strain, DV-1 43251 (genotype I), KP406802 (genotype I), KP406803 (genotype IV), and KP406801 (genotype V), was 10.8, 10.8, 21.4, and 16.9 years, respectively. The TMRCA of the DV-4 epidemic strain containing 43257 (genotype II) was 22 years. Genotype I of Korean DV-1 probably descended from a common ancestor that existed in Singapore during the period 2000–2006. KP406803, belonging to genotype IV, formed a monophyletic group with the most probable ancestral origin in the Philippines and Hawaii during 1997–2006. The genotype V isolate from Korea (KP406801) is estimated to have emerged at least 42.3 years ago. The TMRCA estimates and 95% HPD intervals calculated from the Bayesian coalescent phylogenetic analysis were as follows: DV-4 genotype I, 84.4 years ago; genotype II, 83.4 years ago; genotype III, 112.0 years ago.

### Selection pressure

Selection pressure was analyzed for the structural and non-structural proteins of the DV-1 and DV-4 strains (Tables 5 and 6). The sites under positive or negative selection were identified based on statistical verification using at least two methods. Positive selection in DV-1 strains was observed in the C, prM, NS2A, and NS5 proteins, whereas positive selection in DV-4 strains was observed only in the non-structural proteins NS2A, NS3, and NS5. DV-4 had higher overall rates of negative selection than DV-1 in most structural and non-structural proteins.

**Table 5:** Selection pressure analysis of DV-1

Type of selection pressure		Structural proteins			Non-structural proteins						
		C (114)	prM (166)	E (495)	NS1 (352)	NS2A (218)	NS2B (128)	NS3 (618)	NS4A (127)	NS4B (246)	NS5 (900)
Positive	Number	1	2	0	0	1	0	0	0	0	3
	(%)	(0.9)	(1.2)	(0)	(0)	(0.5)	(0)	(0)	(0)	(0)	(0.3)
Negative	Number	22	70	203	146	94	60	281	61	115	397
	(%)	(19.3)	(42.2)	(41.0)	(41.5)	(43.1)	(46.9)	(45.5)	(48.0)	(46.7)	(44.1)

C, capsid; prM, pre-membrane; E, envelope; NS, non-structural

**Table 6:** Selection pressure analysis of DV-4

Type of selection pressure		Structural proteins			Non-structural proteins						
		C (114)	prM (166)	E (495)	NS1 (352)	NS2A (218)	NS2B (128)	NS3 (618)	NS4A (127)	NS4B (246)	NS5 (900)
Positive	Number	0	0	0	0	1	0	1	0	0	3
	(%)	(0)	(0)	(0)	(0)	(0.5)	(0)	(0.2)	(0)	(0)	(0.3)
Negative	Number	51	121	360	251	160	98	487	100	192	649
	(%)	(44.7)	(72.9)	(72.7)	(71.3)	(73.4)	(76.6)	(78.8)	(78.7)	(78.0)	(72.1)

C, capsid; prM, pre-membrane; E, envelope; NS, non-structural

## Discussion

The incidence of global dengue outbreaks is growing at a very rapid rate, with a 400% increase in recent decades due to global warming, urbanization, and increased global travel [26]. Since 2010, the influx of global travelers infected with DV has been increasing rapidly in Korea [27], putting Korea at high risk for epidemics of dengue disease. Therefore, DV must be continually monitored and characterized to cope with this impending threat. In this study, we conducted the first molecular, biological, and evolutionary analysis of DV-1 and DV-4 isolates from Korea.

The DV-1 strains were previously classified into five distinct genotypes (I–V) [28, 29]. Phylogenetic analysis based on the E protein and the complete coding region revealed that the DV-1 isolates in Korea belong to genotype I (43251, KP406802), genotype IV (KP406803), and genotype V (KP406801) (Fig 1a). The strains D1/Singapore/ EU081276/2005 and D1/Indonesia/KC762641/2008, which were the ones most similar to two Korean DV-1 isolates (43251 and KP406802; genotype I), were the major strains that caused dengue outbreaks in Singapore in 2005 and Indonesia in 2007, respectively [30–32]. Our results indicate that these viruses are still circulating in Southeast Asian countries more than 10 years later. The KP406803 isolate was grouped into the genotype IV clade, which also includes D1/Philippines/GQ868602/2004 and D1/Hawaii/DQ672564/2001. Genotype IV of DV-1 was first isolated in the Philippines in 1974, and it has predominated in each epidemic in the South Pacific since 2000 [33]. Among the five DV-1 genotypes, genotype V has been reported to be the most virulent and to be strongly associated with DHF [9]. We identified one strain (KP406801) from this virulent genotype. As shown by our results, various DV-1 genotypes that have caused epidemics in other countries have been introduced into Korea, where they could cause disease outbreaks.

The substitution rate per site per year of the E protein in DV-1 was 5.58 × 10^-4^, which is high for an RNA virus. The substitution rates for RNA viruses generally range from 10^-6^ to 10^-4^. The rate for DV-1 was similar to that reported previously for DV-2 strains isolated in Korea (5.32 × 10^-4^) [34]. However, the isolation years differed significantly between the Korean DV-1 strains and the most similar strains, differing by 7 to 8 years, even though these strains shared a high degree of nucleotide sequence similarity (98.7–99.5% identity) (Fig. 2a). The average TMRCAs of the DV-1 isolates from this study were 11 (43251 and KP406802), 21 (KP406803), and 17 years (KP406801) [23]. The TMRCA of previously reported DV-2 isolates from Korea (4 to 10 years) was shorter than those of the DV-1 and DV-4 strains we tested, although the overall substitution rates of the DV-2 strains were similar to those in this report [34]. These results suggest that some DV-1 genotypes with slow substitution rates caused concomitant DV outbreaks in Southeast Asia, including in Singapore, and were then introduced into Korea [32, 35].

Among the three genotypes (I–III) of the DV-4 serotype, the Korean DV-4 isolates belong to genotype I (KP406806) and genotype II (43257) (Fig. 1b). The strain most similar to KP406806 is D4/Philippines/AY947539/1956, a representative strain that has been found consistently in China and Taiwan from about 60 years ago until the 2000s. The DV-4 43257 strain, which was isolated from a Korean traveling to the Philippines in 2010, has the highest nucleotide sequence similarity to DV-4 strains circulating in Singapore in 2005. DV-4 genotype II is the predominant genotype in Singapore, indicating that it was introduced into Korea via the Philippines [32]. The substitution rate per site per year of the DV-4 strains was 6.72 × 10^-4^, which was as fast as that of previously reported DV-1 and DV-2 strains [34]. The TMRCA analysis showed that this strain evolved within 20 years. Like the DV-1 isolates, DV-4 strains have been consistently circulating in Southeast Asia without significant mutation for a long time, even though we found DV-4 to have a rapid substitution rate, similar to those of DV-1 and DV-2 in Korea.

Several amino acid sequence motifs affecting viability and virulence have been found in DV proteins. An S112A substitution in the prM protein inhibits the assembly of replicon particles, and a T193A mutation in the E protein reduces the infectivity of DV-1 tenfold [24]. An R888 mutation results in incorrect localization of NS5, affecting the synthesis of negative-stranded RNA [25]. A 40-aa deletion in the DV-4 NS2B protein eliminates autoproteolytic activity [36]. A D192N substitution in the NS3 protein causes increased neurovirulence in mice [37]. However, we did not find any amino acid substitution mutations known to be related to virulence or viability in the Korean DV-1 and DV-4 isolates we studied (Supplementary Tables S2 and S3).

Compared with EU848545 and AF326573, non-synonymous substitutions were observed in an average of 6.7 and 5 sites in the structural proteins of DV-1 and DV-4, respectively (Tables 3 and 4). In particular, amino acid mutations in the E protein can lead to structural changes in cell binding and immunogenicity [38], possibly leading to immune escape in the host [39]. In a previous report, sites near epitopes in the hemagglutinin of influenza viruses known to be important for antigenicity were mainly under positive selection, which favors non-synonymous substitutions [40]. The C and prM structural proteins and NS2A and NS5 non-structural proteins in DV-1 were found to be under positive selection (Table 5). However, positive selection in DV-4 was found only in the NS2A, NS3, and NS5 non-structural proteins (Table 6). NS2A and NS5 play important roles in viral RNA replication and the suppression of host immune responses [41, 42]. Therefore, those proteins might be under positive pressure to escape host immunity. On the other hand, the E protein was not found to be under positive selection in the Korean DV-1 or DV-4 strains, even though the E protein of DV is known to be important for immunogenicity. This finding is consistent with that of previous studies showing that the E protein of DV-1 and DV-2 was not strongly affected by positive selection [34, 43]. The role of the proteins under positive pressure should be evaluated carefully to prepare for future DV epidemics. Interestingly, significantly fewer sites in DV-1 were under negative selective pressure than in DV-2 and DV-4. This significant difference suggests that the DV-1 strains have a more stable evolutionary status than the DV-2 and DV-4 strains [44].

In this study, we performed molecular and evolutionary analysis of DV-1 and DV-4 isolates from Korea. Because many global travelers with dengue infection are asymptomatic or have mild symptoms, the actual incidence of DV infection in Koreans is probably higher than the number of reported cases. Nevertheless, considering the extremely limited amount of information that was previously available about DV-1 and DV-4 isolates in Korea, this study has provided the first comprehensive molecular, phylogenetic, and evolutionary information about DV-1 and DV-4 strains in Korea. Various genotypes of DV-1 and DV-4 with distinct characteristics have been introduced into Korea, and some of them could cause autochthonous outbreaks in Korea. Therefore, further characterization and surveillance of DV should be performed.

## Supplementary Information

Below is the link to the electronic supplementary material.

Supplementary file1 (DOCX 22 KB)

## References

[1] Wilder-Smith A, Ooi EE, Horstick O, Wills B (2019). Dengue. Lancet.

[2] Guzman MG, Harris E (2015). Dengue. Lancet.

[3] Guo C, Zhou Z, Wen Z, Liu Y, Zeng C, Xiao D, Ou M, Han Y, Huang S, Liu D (2017). Global epidemiology of dengue outbreaks in 1990–2015: a systematic review and meta-analysis. Front Cell Infect Microbiol.

[4] Ramos-Castaneda J, dos Santos FB, Martinez-Vega R, de Araujo JMG, Joint G, Sarti E (2017). Dengue in Latin America: systematic review of molecular epidemiological trends. PLoS Negl Trop Diseases.

[5] Struchiner CJ, Rocklöv J, Wilder-Smith A, Massad E (2015). Increasing dengue incidence in Singapore over the past 40 years: population growth, climate and mobility. PLoS ONE.

[6] Shepard DS, Undurraga EA, Halasa YA (2013). Economic and disease burden of dengue in Southeast Asia. PLoS Negl Trop Dis.

[7] Organization WH (2014) Dengue and severe dengue. World Health Organization. Regional Office for the Eastern Mediterranean

[8] Lambrechts L, Fansiri T, Pongsiri A, Thaisomboonsuk B, Klungthong C, Richardson JH, Ponlawat A, Jarman RG, Scott TW (2012). Dengue-1 virus clade replacement in Thailand associated with enhanced mosquito transmission. J Virol.

[9] Rico-Hesse R (2003). Microevolution and virulence of dengue viruses. Adv Virus Res.

[10] Srikiatkhachorn A, Mathew A, Rothman AL (2017). Immune-mediated cytokine storm and its role in severe dengue. Semin Immunopathol.

[11] Zou C, Huang C, Zhang J, Wu Q, Ni X, Sun J, Dai J (2019). Virulence difference of five type I dengue viruses and the intrinsic molecular mechanism. PLoS Negl Trop Dis.

[12] Prestwood TR, Prigozhin DM, Sharar KL, Zellweger RM, Shresta S (2008). A mouse-passaged dengue virus strain with reduced affinity for heparan sulfate causes severe disease in mice by establishing increased systemic viral loads. J Virol.

[13] Cho K-h, Park S-Y, Lee W-C, Lee M-J, Lee J-b (2018). International travel and exotic dengue fever in South Korea from 2006 to 2015. Jpn J Infect Dis.

[14] Jeong YE, Lee WC, Cho JE, Han MG, Lee WJ (2016). Comparison of the Epidemiological Aspects of Imported Dengue Cases between Korea and Japan, 2006–2010. Osong Public Health Res Perspect.

[15] Lee H, Kim JE, Lee S, Lee CH (2018). Potential effects of climate change on dengue transmission dynamics in Korea. PLoS ONE.

[16] Park G-H, Kim SI, Cho SW, Cho S-R, Lee S-J, Kim HK, Koo H-N, Lee W-G, Cho S-H, Kim G-H (2018). Seasonal distribution of mosquitoes according to habitat environment (2016–2018). Korean J Appl Entomol.

[17] Yung C-F, Lee K-S, Thein T-L, Tan L-K, Gan VC, Wong JGX, Lye DC, Ng L-C, Leo Y-S (2015). Dengue serotype-specific differences in clinical manifestation, laboratory parameters and risk of severe disease in adults, Singapore. Am J Trop Med Hyg.

[18] Nisalak A, Endy TP, Nimmannitya S, Kalayanarooj S, Scott RM, Burke DS, Hoke CH, Innis BL, Vaughn DW (2003). Serotype-specific dengue virus circulation and dengue disease in Bangkok, Thailand from 1973 to 1999. Am J Trop Med Hyg.

[19] Cruz CD, Torre A, Troncos G, Lambrechts L, Leguia M (2016). Targeted full-genome amplification and sequencing of dengue virus types 1–4 from South America. J Virol Methods.

[20] Kumar S, Stecher G, Li M, Knyaz C, Tamura K (2018). MEGA X: molecular evolutionary genetics analysis across computing platforms. Mol Biol Evol.

[21] Weaver S, Shank SD, Spielman SJ, Li M, Muse SV, Kosakovsky Pond SL (2018). Datamonkey 2.0: a modern web application for characterizing selective and other evolutionary processes. Mol Biol Evol.

[22] Dash PK, Sharma S, Soni M, Agarwal A, Sahni AK, Parida M (2015). Complete genome sequencing and evolutionary phylogeography analysis of Indian isolates of Dengue virus type 1. Virus Res.

[23] Yohan B, Wardhani P, Trimarsanto H, Aryati A, Sasmono RT (2018). Genomic analysis of dengue virus serotype 1 (DENV-1) genotypes from Surabaya, Indonesia. Virus Genes.

[24] Ahmad Z, Poh CL (2019). The conserved molecular determinants of virulence in dengue virus. Int J Med Sci.

[25] Tay MY, Smith K, Ng IH, Chan KW, Zhao Y, Ooi EE, Lescar J, Luo D, Jans DA, Forwood JK, Vasudevan SG (2016). The C-terminal 18 amino acid region of Dengue Virus NS5 regulates its subcellular localization and contains a conserved arginine residue essential for infectious virus production. PLoS Pathog.

[26] Fitzmaurice C, Allen C, Barber RM, Barregard L, Bhutta ZA, Global Burden of Disease Cancer C (2017). Global, Regional, and National Cancer Incidence, Mortality, years of life lost, years lived with disability, and disability-adjusted life-years for 32 Cancer Groups, 1990 to 2015: a systematic analysis for the global burden of disease study. JAMA Oncol.

[27] Miki S, Lee W-C, Lee M-J (2017). A comparative study of the trends of imported Dengue cases in Korea and Japan 2011–2015. J Clin Med Res.

[28] Goncalvez AP, Escalante AA, Pujol FH, Ludert JE, Tovar D, Salas RA, Liprandi F (2002). Diversity and evolution of the envelope gene of dengue virus type 1. Virology.

[29] Teoh B-T, Sam S-S, Abd-Jamil J, AbuBakar S (2010). Isolation of ancestral sylvatic dengue virus type 1, Malaysia. Emerg Infect Dis.

[30] Low JG, Ooi E-E, Tolfvenstam T, Leo Y-S, Hibberd ML, Ng L-C, Lai Y-L, Yap G, Li C, Vasudevan SG (2006). Early Dengue infection and outcome study (EDEN)-study design and preliminary findings. Ann Acad Med Singap.

[31] Sasmono RT, Wahid I, Trimarsanto H, Yohan B, Wahyuni S, Hertanto M, Yusuf I, Mubin H, Ganda IJ, Latief R (2015). Genomic analysis and growth characteristic of dengue viruses from Makassar, Indonesia. Infect Genet Evol.

[32] Lee K-S, Lo S, Tan SS-Y, Chua R, Tan L-K, Xu H, Ng L-C (2012). Dengue virus surveillance in Singapore reveals high viral diversity through multiple introductions and in situ evolution. Infect Genet Evol.

[33] Sun Y, Meng S (2013). Evolutionary history and spatiotemporal dynamics of dengue virus type 1 in Asia. Infect Genet Evol.

[34] Hwang EH, Kim G, Oh H, An YJ, Kim J, Kim JH, Hwang ES, Park JH, Hong J, Koo BS (2020). Molecular and evolutionary analysis of dengue virus serotype 2 isolates from Korean travelers in 2015. Arch Virol.

[35] Koo C, Tien WP, Xu H, Ong J, Rajarethinam J, Lai YL, Ng L-C, Hapuarachchi HC (2018). Highly selective transmission success of dengue virus type 1 lineages in a dynamic virus population: an evolutionary and fitness perspective. iScience.

[36] Falgout B, Miller RH, Lai CJ (1993). Deletion analysis of dengue virus type 4 nonstructural protein NS2B: identification of a domain required for NS2B-NS3 protease activity. J Virol.

[37] Blaney JE, Johnson DH, Manipon GG, Firestone CY, Hanson CT, Murphy BR, Whitehead SS (2002). Genetic basis of attenuation of dengue virus type 4 small plaque mutants with restricted replication in suckling mice and in SCID mice transplanted with human liver cells. Virology.

[38] Zhang X, Jia R, Shen H, Wang M, Yin Z, Cheng A (2017). Structures and functions of the envelope glycoprotein in flavivirus infections. Viruses.

[39] Tazeen A, Afreen N, Abdullah M, Deeba F, Haider S, Kazim S, Ali S, Naqvi I, Broor S, Ahmed A (2017). Occurrence of co-infection with dengue viruses during 2014 in New Delhi, India. Epidemiol Infect.

[40] Duvvuri VRSK, Duvvuri B, Cuff WR, Wu GE, Wu J (2009). Role of positive selection pressure on the evolution of H5N1 hemagglutinin. Genom Proteom Bioinform.

[41] De Maio FA, Risso G, Iglesias NG, Shah P, Pozzi B, Gebhard LG, Mammi P, Mancini E, Yanovsky MJ, Andino R, Krogan N, Srebrow A, Gamarnik AV (2016). The Dengue Virus NS5 protein intrudes in the cellular spliceosome and modulates splicing. PLoS Pathog.

[42] Xie X, Gayen S, Kang C, Yuan Z, Shi P-Y (2013). Membrane topology and function of dengue virus NS2A protein. J Virol.

[43] Patil J, Cherian S, Walimbe A, Patil B, Sathe P, Shah P, Cecilia D (2011). Evolutionary dynamics of the American African genotype of dengue type 1 virus in India (1962–2005). Infect Genet Evol.

[44] Behura SK, Severson DW (2013). Nucleotide substitutions in dengue virus serotypes from Asian and American countries: insights into intracodon recombination and purifying selection. BMC Microbiol.

