# Supplementary material for: Molecular evolution of dengue virus types 1 and 4 in Korean travelers
Source: Arch Virol. 2021 Feb 11;166(4):1103–12. doi: 10.1007/s00705-021-04973-8 (PMC7952331; doi:10.1007/s00705-021-04973-8)
Supplement: Supplementary file 1 — Supplementary file1 (DOCX 22 KB) [file 705_2021_4973_MOESM1_ESM.docx]

**Table S1** Primer pairs targeting the full coding region of DV-1 and DV-4

| Primer name | Sequence | Nucleotide position | Reference |
| --- | --- | --- | --- |
| d1f1 | ACGTGGACCGACAAGAACAGTTTCG | 13-37 | Cruz et al. |
| d1f2 | AACAACCAACGGAAAAAGAC | 98-117 | This study |
| d1r21 | CYTTCTTGAACCAGCTTAGT | 2098-2117 | This study |
| d1f11 | ATTCGAGATGTCCAACACAA | 1146-1165 | This study |
| d1r33 | TCTCCTYTGAAACGTAAGGG | 3396-3377 | This study |
| d1f29 | RTGAAATTGCGTGACTCCTA | 2924-2943 | This study |
| d1r55 | CTCATCTTGGATARCTGCRT | 5508-5527 | This study |
| d1f45 | ACAGAGATCAGGAGTGYTRT | 4513-4532 | This study |
| d1r67 | RCACCATCAGRAAGAACTCC | 6709-6728 | This study |
| d1f61 | ATCCTACAAAGTTGCCTCAG | 6160-6179 | This study |
| d1r90 | TGCTGAACCARTGRTCTTCR | 9049-9068 | This study |
| d1f88 | AGGGAGCTTCAYAARCAAGG | 8885-8904 | This study |
| d1r107 | CGTTCTGTGCCTGGAATGATGCTG | 10684-10707 | Cruz et al. |
| d4f1 | AGTTGTTAGTCTGTGTGGACCGACAA | 01~26 | Cruz et al. |
| d4f2 | CGACAAGGACAGTTCCAAAT | 19-38 | This study |
| d4r27 | TTTCAGRTCATTCACTGGRG | 2738-2757 | This study |
| d4f21 | TAACACTCCATTGGTTCAGR | 2096-2115 | This study |
| d4r44 | GGAGGGTWATCATRTTGGTT | 4404-4423 | This study |
| d4f42 | GAATGATGTCCCTCTRGCTG | 4200-4219 | This study |
| d4r59 | TACTGGYCATCTTCTTGYGC | 5917-5936 | This study |
| d4f54 | GAGGCWGCAGCYATCTTTAT | 5443-5462 | This study |
| d4r82 | CYGAYACACYTGACACCCAA | 8217-8236 | This study |
| d4f74 | CATTTTCAGGGGAAGCTAYT | 7485-7504 | This study |
| d4r104 | TAACCGCTAGTCCAATATGC | 10407-10426 | This study |
| d4r106 | AGAACCTGTTGGATCAACAACACCAAT | 10630-10650 | Cruz et al. |

**Table S2** Amino acid mutations in DV-1 sequences that contribute to virulence or viability

| Protein | Position | Strain | | | | |
| --- | --- | --- | --- | --- | --- | --- |
|  |  | Virulence | 43251 | KP406801 | KP406802 | KP406803 |
| prM | 112 | S→A | S | S | S | S |
| E | 193 | T→A | T | T | T | T |
| NS2A | 181 | L→F | L | L | L | L |
| NS5 | 884 | P→T | T | T | T | T |
| NS5 | 888 | R→A | R | R | R | R |
| NS5 | 888 | R→E | R | R | R | R |
| NS5 | 888 | R→K | R | R | R | R |
| NS5 | 330 | K→A | K | K | K | K |

prM, pre-membrane; E, envelope; NS, non-structural

**Table S3** Amino acid mutations in DV-4 sequences that contribute to virulence or viability

| Protein | Position | Strain | | |
| --- | --- | --- | --- | --- |
|  |  | Virulence | 43257 | KP406806 |
| NS2B | 54-92 | 40 amino acid deletion | AA | AA |
| NS3 | 192 | D→N | D | D |
| NS5 | 884 | P→T | P | P |
| NS5 | 888 | R→A | R | R |

NS, non-structural
